# Genome segment 5 of *Antheraea mylitta* cytoplasmic polyhedrosis virus encodes a bona fide guanylyltransferase

**DOI:** 10.1186/1743-422X-11-53

**Published:** 2014-03-21

**Authors:** Poulomi Biswas, Anirban Kundu, Ananta Kumar Ghosh

**Affiliations:** 1 Department of Biotechnology, Indian Institute of Technology Kharagpur, Kharagpur 721302, India

**Keywords:** *Antheraea mylitta*, Cytoplasmic polyhedrosis virus, Genome segment 5, Guanylyltransferase, Kinetic and thermodynamic analysis, Cloning and sequencing, Expression

## Abstract

**Background:**

*Antheraea mylitta* cytoplasmic polyhedrosis virus (AmCPV), a cypovirus of *Reoviridae* family, infects non mulberry Indian silk worm, *Antheraea mylitta,* and contains eleven segmented double stranded RNA in its genome (S1-S11). Some of its genome segments (S1-S3, and S6-S11) have been previously characterized but genome segment encoding the viral guanylyltransferase which helps in RNA capping has not been characterized.

**Results:**

In this study genome segment 5 (S5) of AmCPV was converted to cDNA, cloned and sequenced. S5 consisted of 2180 nucleotides, with one long ORF of 1818 nucleotides and could encode a protein of 606 amino acids with molecular mass of ~65 kDa (p65). Bioinformatics analysis showed presence of KLRS and HxnH motifs as observed in some other reoviral guanylyltransferase and suggests that S5 may encodes viral guanylyltransferase. The ORF of S5 was expressed in *E. coli* as 65 kDa his tagged fusion protein, purified through Ni-NTA chromatography and polyclonal antibody was raised. Immunoblot analysis of virion particles with the purified antibody showed specific immunoreactive band and suggests p65 as a viral structural protein. Functional analysis showed that recombinant p65 possesses guanylyltransferase activity, and transfers GMP moiety to the 5' diphosphate (A/G) ended viral RNA after the formation of p65-GMP complex for capping. Kinetic analysis showed K_m_ of this enzyme for GTP and RNA was 34.24 uM and 98.35 nM, respectively. Site directed mutagenesis at K21A in KLRS motif, and H93A or H105A in HxnH motif completely abolished the autoguanylylation activity and indicates importance of these residues at these sites. Thermodynamic analysis showed p65-GTP interaction was primarily driven by enthalpy (ΔH = -399.1 ± 4.1 kJ/mol) whereas the p65-RNA interaction by favorable entropy (0.043 ± 0.0049 kJ/ mol).

**Conclusion:**

Viral capping enzymes play a critical role in the post transcriptional or post replication modification in case of RNA virus. Our results of cloning, sequencing and functional analysis of AmCPV S5 indicates that S5 encoded p65 through its guanylyltransferase activity can transfer guanine residue to the 5' end of viral RNA for capping. Further studies will help to understand complete capping process of cypoviral RNA during viral replication within the viral capsid.

## Background

Cytoplasmic polyhedrosis virus (CPV) belongs to the genus Cypovirus of the family *Reoviridae *[1] and contains 10 segmented double stranded (ds) RNA [2]. Each ds RNA segment is composed of a plus strand and it’s complementary minus strand in end to end base pair configuration except for a protruding 5′ capped end [3]. CPV virions embedded in polyhedra are capable of surviving dehydration, freezing, and chemical treatment that would denature most proteins [4]. In the alkaline mid gut of insect hosts, polyhedra dissolve and release virions to infect intestinal epithelial cells. Cypovirus is unique among the dsRNA viruses in having a single capsid layer which functions as a stable mRNA synthesis machine to transcribe 5′ capped mRNA and also serves as shelter protecting this transcriptional process from anti-viral defence mechanism present inside the cytoplasm of host cells [5].

Generally in reovirus, the core synthesizes, modifies and exports viral mRNA. The RNA capping and export apparatus look like a hollow cylinder which probably sequesters the nascent mRNA to be capped [6]. The capping of nascent viral RNA strand is mediated by a guanylyltransferase (GTase) that uses GTP to form a covalently bound enzyme-substrate intermediate (autoguanylylation) before the GMP residue is transferred to 5′ end of mRNA. In case of CPV infecting mulberry silk worm, *Bombyx mori* (BmCPV), capsid is formed by three major proteins: VP1 (capsid shell protein), VP3 (turret protein) and VP5 (spike like protein) encoded by its genome segment 1, 4 and 7, respectively. Analysis of three dimensional structure of BmCPV by cryo electron microscopy reveals that the slanted disposition of turret protein functional domains and the stacking of channel constrictions creates an iris diaphragm like mechanism for opening/closing of capsid pores and turret channels in regulating the highly coordinated steps of mRNA transcription, processing and release [7]. Thus in BmCPV which belongs to the subgroup of “turreted reovirus”, mRNA capping occurs in a pentameric turret whose five unique channels guide nascent mRNA sequentially to GTase, N-7 methyl transferase and 2′–O-methyl transferase domains in order to fulfil highly coordinated mRNA capping activity [7,8]. The most recent structural comparison via cryo-EM of transcribing and non transcribing BmCPV showed that transfer of GMP moiety occurs to the 5′ end of the di phosphate ended RNA after its binding to the Lys^234^ residue of GTase pocket via phosphoamide linkage [9].

In case of viruses lacking pentameric turret, core protein VP3 (in case of rotavirus) or VP4 (in case of bluetongue virus) acts as GTase to react with GTP for the formation of GMP-enzyme intermediate via phospohoamide linkage for the transfer of GMP to the 5′ diphosphate ended viral RNA. Recent structural study on rotavirus showed that RNA dependent RNA polymerase (VP1) and RNA capping enzyme (VP3) associated with each genome segment are anchored to the interior side of the capsid (VP2) through contacts made near the fivefold axis [10].

A detailed thermodynamic study on the interaction of *Saccharomyces cerevisiae* RNA GTase (Ceg1) with GTP, RNA and manganese ions reveals that the initial association of GTP with the Ceg1 protein is driven by a favorable enthalpy change and the interaction between Ceg1 and RNA is clearly determined by a favourable entropic effect [11].

*Antherea mylitta* cytoplasmic polyhedrosis virus (AmCPV) is a big threat to Indian non-mulberry silkworm, *Antheraea mylitta,* destroying about 30% crop each year [12]. AmCPV genome consists of 11 ds segmented RNA (S1-S11) [13]. Most of these genome segments except S4 and S5 have been cloned, sequenced, and functions of some of them have been determined. S1 and S3 code for viral capsid proteins [14], S2 codes for RNA dependent RNA polymerase [15], S6 codes for protein having ATP binding and ATPase activity [16], S7 and S8 encode viral structural proteins [17,18], S9 codes for a non-structural protein having RNA binding property [19]. S10 codes for viral polyhedron [20], and S11 does not contain any ORF [18]. To determine the role of S5 encoded protein in viral life cycle it is necessary to clone and characterize this genome segment. Since sequences of different genome segments of AmCPV do not show any homology with BmCPV or other cypoviral sequences, and the mechanism of capping of the 5′ end of AmCPV RNA has not been reported, molecular, biochemical and thermodynamical characterization of AmCPV capping enzymes are very much necessary.

Here we report molecular cloning, sequencing and expression of AmCPV genome segment 5 (S5) and show that it encodes a 65 kDa protein (p65). Using biochemical assays we show that p65 is a bona fide guanylyltransferase which catalyzes formation of RNA cap structure using GTP as substrate through initial formation of enzyme-GMP complex via phosphoamide linkage and then transferring the GMP moiety to diphosphate ended viral RNA. By performing thermodynamic analysis we also show that p65-GTP interaction is primarily driven by enthalpy and its binding to RNA is dominated by entropy.

## Results and Discussion

### Genetic analysis of AmCPV S5

**Figure 1:**
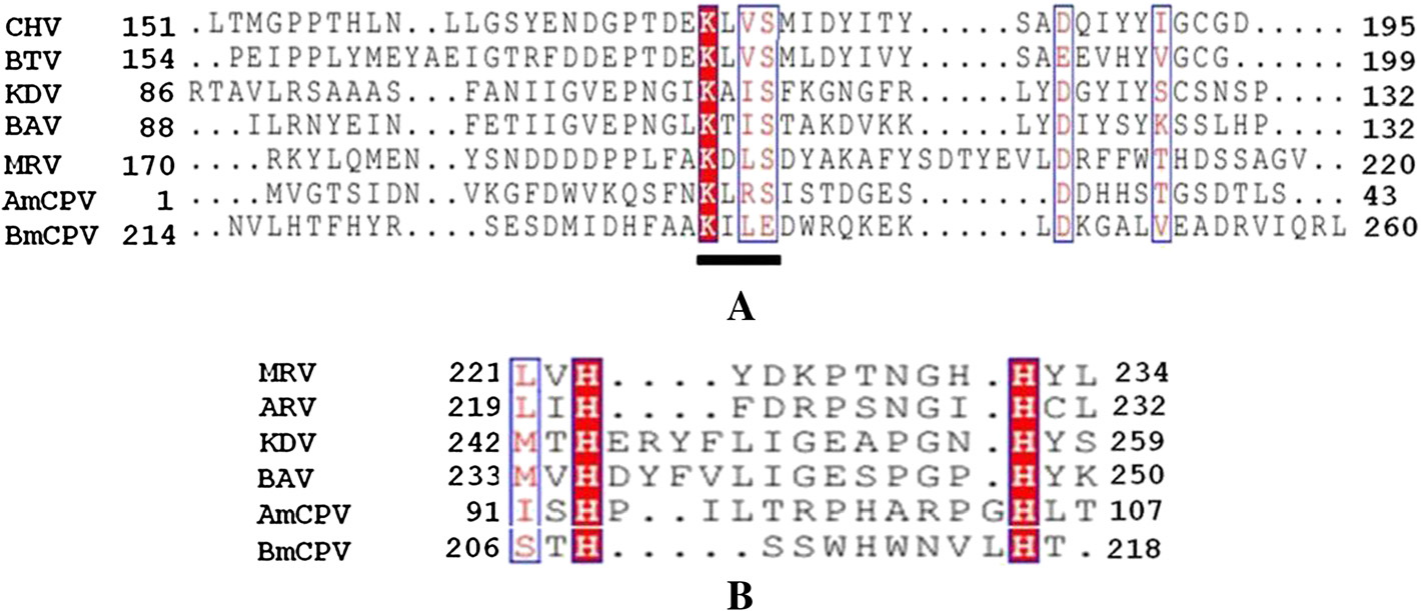
**Amino acid sequence alignment of AmCPV S5 protein with the guanylyltransferases of other reoviruses. (A)** in the vicinity of putative Kx(I/V/L/R) S (bold black underlined) motif. [CHV (accession no: BAA76550), BAV (accession no:NP_694476); KDV (accession no; NP_694471); BTV (accession no: AF173127); ARV (accession no: AAM46173); MRV (accession no: NP_694680); AmCPV S5 (accession no: JX853836); BmCPV (accession no: ADB95943)] and **(B)** in the vicinity of putative (HxnH) motif.

AmCPV S5 ds RNA was isolated, reverse transcribed to its cDNA, cloned into pCR-XL-Topo vector and sequenced. The AmCPV S5 cDNA consisted of 2180 nucleotides with a single ORF of 606 amino acid residues which could encode a protein of ~65 kDa (p65). The S5 ORF started with an ATG codon at 266^th^ nucleotide and ended with the TAA stop codon at 2086^th^ nucleotide. It is interesting to note that although in all other segments of AmCPV the initiation codon lies within 40 nucleotides from the 5′ end but in S5 it is located at 266^th^ nucleotide. This is somehow unusual for CPV although Reuter et al. [21] and Graham et al. [22] have reported presence of 171 bp and 141 nt long 5′UTR sequence in segment 6 of a novel seadornavirus related to Banna virus in Europe and in segment 5 of Choristoneura *occidentalis* CPV16, respectively. The theoretical isoelectric point of p65 was calculated as 6.14. The protein is found rich in leucine, serine, isoleucine, threonine, and arginine amino acid residues. The secondary structure prediction using GOR4 showed that 51.98% of residues are likely to form random coils, 22.11% would form α-helices, and 25.91% would form extended sheets. BLAST analysis did not show any significant homology of p65 with any other protein sequences in the public databases. Therefore, to assess the possible role of p65 in viral life cycle, deduced amino acid sequences of p65 were analyzed with known conserved motifs of several ds RNA viral proteins. This type of analysis showed that p65 could be aligned with GTase domain (Figure 1A) containing a conserved Kx(I/V/L)S and HxnH motifs of Chuzan virus (CHV), blue tongue virus (BTV), Kadipiro virus (KDV), avian orthoreovirus (ARV), Banna virus (BAV), mammalian reovirus (MRV) and BmCPV [23,24], and indicated that S5 may codes for GTase enzyme which helps in the capping of 5′ end of viral RNA. The analysis also showed that GTase domain of other dsRNA viruses contained Kx(I/V/L)S motif whereas AmCPV p65 contained KxRS motif at position 21 to 24 amino acid residues. Replacement of a hydrophobic amino acid residue with a positively charged arginine residue in p65 may impose a structure of its unique GTase activity. The lysine residue at position 21 of KLRS motif of p65 was found conserved as observed among other members of the *Reoviridae* family and suggested to be involved in phosphoamide bond formation with GMP moiety [25]. Secondary structure analysis of p65 showed that lysine residue of KLRS motif was located in a loop (data not shown) as observed in orthoreovirus GTase [6], but is different from BmCPV GTase where the conserved lysine residue of its KILE motif was located in an alpha-helix [9]. It has also been observed that AmCPV p65 contained one putative HxnH motif between amino acid residues 93 and 105 as found in other dsRNA viral GTase [23] for interacting with phosphate group of GTP during autoguanylylation and capping reaction (Figure 1B). Amino acid sequence alignment also showed that except for KDV and BAV (where two histidinies are located twelve residues apart) the distance between two histidine residues are eight amino acids in case of BmCPV, ARV and MRV. In comparison to these, in AmCPV two histidine residues in this motif are located seven amino acids apart (Figure 1B). Secondary structure analysis also allowed us to predict H93 and H105 as active histidine pair in p65 because they were found in a loop position flanked by two β strands as observed in case of BmCPV and orthoreovirus [7,9] suggesting again that p65 may code for GTase. In BmCPV GTase, the HxnH motif is present 27 residues before the KILE motif (between 208 and 217 amino acid residues) but in AmCPV it is located 72 amino acids before KLRS motif and indicates some structural differences between different CPV GTase.

### Expression and purification of recombinant p65

**Figure 2:**
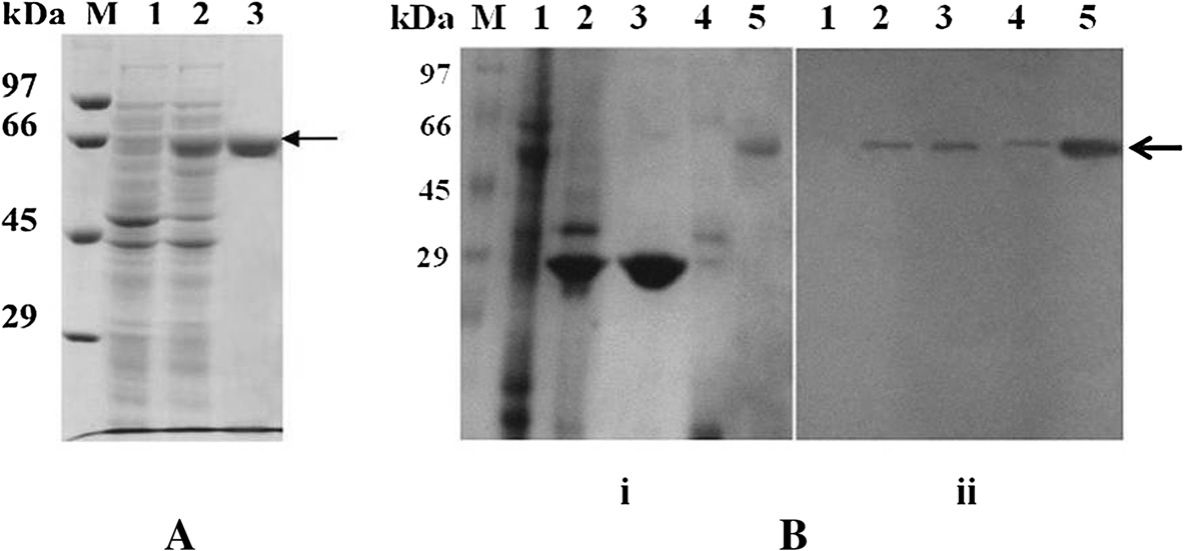
**p65 expression in E. coli and western blotting. (A)** Analysis recombinant p65 by SDS-PAGE. Lane 1, Molecular wt. marker; lane 2, uninduced *E. Coli*; lane 3, IPTG induced *E. coli* and lane 3, purified p65 (~65 kDa). **(B)** Immunoblot analysis of mid gut cells using anti-p65 antibody: (i) SDS-10% PAGE and (ii) western blot. Lane M, Molecular weight marker; lane 1, uninfected midgut; lane 2, infected midgut; lane 3, purified polyhedra; lane 4, virion particle; lane 5, purified recombinant p65. Arrow indicates position of p65.

The entire ORF of AmCPV S5 was expressed in *E. coli* via pQE-30 vector to produce histidine tagged fusion protein. The soluble protein was obtained by sonicating recombinant bacteria in buffer A (10 mM Tris–HCl, 300 mM NaCl, pH 8.0), and purified through Ni-NTA chromatography (Figure 2A). Maximum protein was eluted from the column with buffer A containing 50 mM imidazole. Analysis of purified protein by SDS-PAGE showed a single band of 65 kDa (Figure 2A, lane 3) and indicated purification of recombinant p65 to homogeneity. Polyclonal antibody was raised in rabbit against the recombinant p65 and purified using antigen (p65) affinity chromatography. Purified antibody up to dilution of 1:10,000 was able to detect antigen (p65) by ELISA. These results indicate that p65 is antigenic and the raised antibody can be used to detect the presence of AmCPV in infected silk worms.

### Immunoblot analysis of p65 in the mid gut cells of AmCPV infected larvae

To confirm the presence of the p65 in virion as structural or non-structural protein, immunoblot analysis was done in virus infected and virus uninfected mid gut cells of fifth instar *A. mylitta* larvae using anti-p65 antibody. A major immunoreactive band of approximately 65 kDa was observed in case of infected mid gut cells [Figure 2B (ii), lane 2]; purified polyhedra [Figure 2B (ii), lane 3] and native virion particles [Figure 2B (ii), lane 4]. But no such band was detected in case of uninfected mid gut cells [Figure 2B (ii), lane 1]. This observed immunoreactive band (65 kDa) of infected mid gut cells, polyhedra and purified virus was found to migrate in the same location with purified bacterially expressed recombinant p65 (Figure 2B, lane 5). These results indicate that AmCPV S5 produced a 65 kDa structural protein after translation initiation from 266 nt position of S5 cDNA and may exist in association with capsid to exert its action as has been reported earlier in case of BmCPV [7].

The size of most of the capping enzymes reported so far for different reoviruses lies between 76 to 142 kDa [3,24,26,27]. In this respect the size of the capping enzyme of AmCPV is the smallest one. The bigger size of bluetongue viral VP4 (76 kDa), rotaviral VP3 (84 kDa) and BmCPV VP3 (130 kDa) may be due to presence of GTase, 7-N-methyl transferase (7-N-Mtase) and 2′–O-methyl transferase (2′–O-MTase) activity in the same protein [26,28]. The structural superimposition and the tertiary structure based amino acid sequence alignment between BmCPV VP3 and lambda 2 orthoreovirus reveals that both of them contain four domains: the GTase domain, the bridge domain, the 7-N-MTase domain and 2′–O-MTase domain. In addition, BmCPV VP3 has an extra small domain called brace domain (not found in lambda 2) and braces the spike like complex (formed by segment 7 encoded protein) in the pentameric turret [9]. Since the AmCPV p65 shares no detectable sequence homology with BmCPV or other viruses of *Reoviridae* family we could not predict its structure based on homology modelling. But biochemical assay has shown that AmCPV p65 possess only GTase activity without any MTase activity (data not shown) and to complete the capping reaction MTase activity may be provided by proteins encoded by other genome segment. In case of an insect orbivirus JKT-7400, a dsRNA virus of the *Reoviridae* family, MTase and GTase activity have been reported to exist in different proteins e.g.VP4 encodes MTase [29] and VP6 encodes GTase [30]. Therefore, it is suggested that the smallest size of capping enzyme of AmCPV encoded by S5 may be due to presence of only GTase domain and the required MTase domain is encoded by a different genome segment.

### Covalent binding of recombinant p65 to GTP

**Figure 3:**
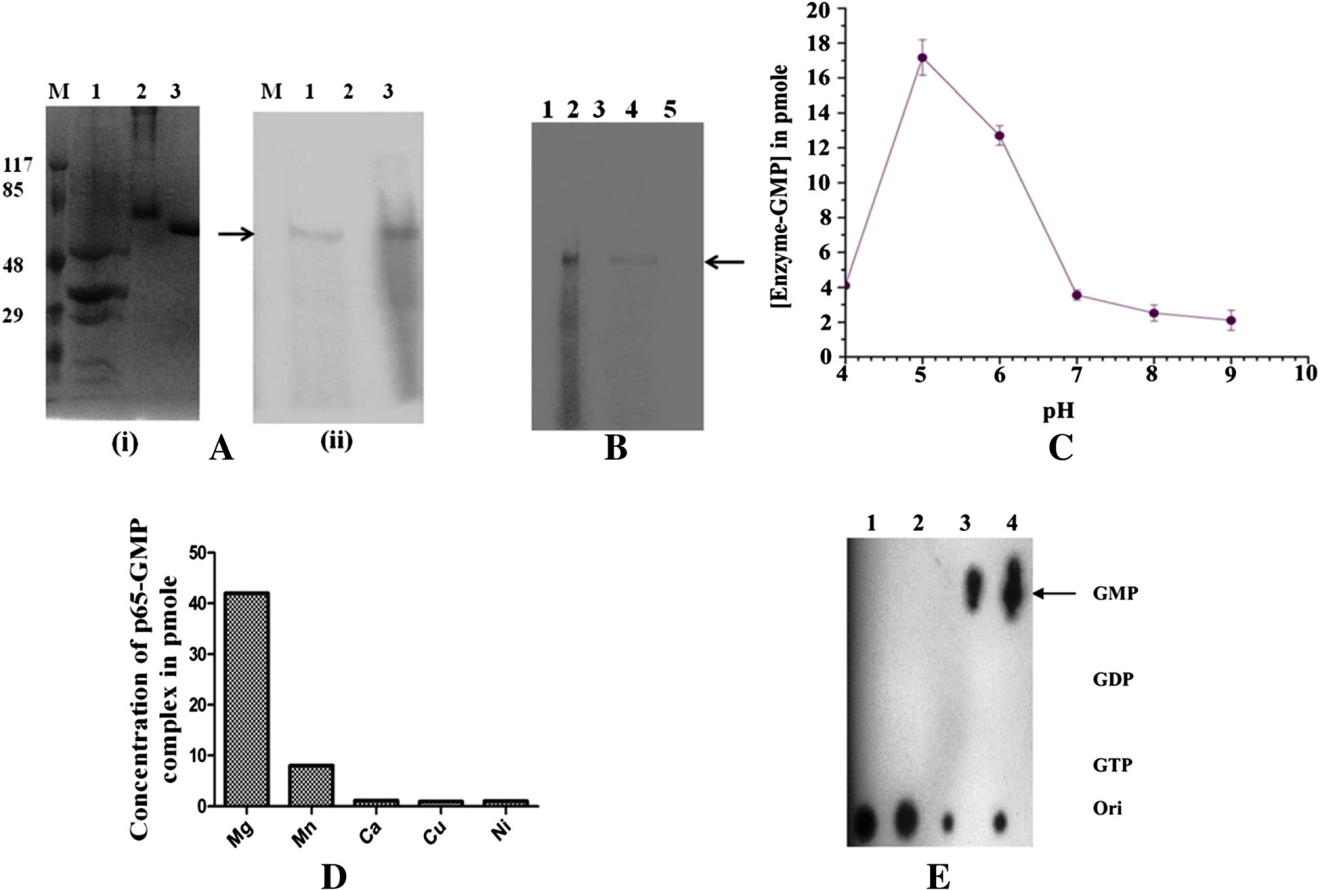
**Interaction of p65 with GMP. (A)** Analysis of the p65–GMP complex formation by autoguanylation (i) SDS-10% PAGE and (ii) autoradiography. Lane M, molecular weight marker; lane 1, virion particle; lane 2, BSA and Lane 3, purified recombinant p65. Arrow indicates the position of the radiolabelled protein band **(B)** Effect of mutations on autoguanylation activity of p65. Lane 1, mutated (K21A) p65 protein; lane 2, wild type p65 protein; lane 3, mutated H93A protein; lane 4, virion particle of AmCPV; lane 5, mutated H105A protein. Arrow indicates the position of the radiolabelled protein band **(C)** Effect of pH on autoguanylation assay. **(D)** Effect of divalent ions in the formation of p65-GMP complex. **(E)** Analysis of the stability of p65-GMP complex by TLC: Lane 1, negative control; lane 2, NaOH treatment; lane 3, HCl treatment; lane 4, NH_2_OH treatment.

The presence of a 5′ cap structure in the plus strand of all the genome segments of cypoviral RNA has been previously reported [3,8]. The first step of the RNA GTase reaction entails the nucleophilic attack of the α-phosphate of GTP leading to the formation of enzyme-GMP intermediate, a process called autoguanylylation [31]. To detect the ability of the purified p65 to form covalent p65-GMP intermediate, purified p65 was incubated with [α-^32^P] GTP at 25°C in presence of Mg^2+^ and the transfer of label from [α-^32^P] GTP to p65 was determined by analysing the product on SDS–10% PAGE followed by autoradiography. A single stable p65-GMP complex was detected in gel [Figure 3A(ii), lane 3] but no such labelled band was observed upon incubation with BSA [Figure 3A(ii), lane 2] indicating autoguanylylation of p65. When similar experiment was performed with virion particle a labelled 65 kDa band was also observed [Figure 3A(ii), lane 1] indicating binding of label to a putative guanylyltransferase in the virions whose molecular weight is 65 kDa.

Biochemical and mutational analysis of the orthoreovirus lambda 2 protein revealed that Lys^190^ in KDLS sequence is necessary for the formation of phosphoamide bond and sequence similar to KDLS is found to be conserved among the RNA GTases of the *Reoviridae* family within the genera retrovirus, orbivirus and phytoreovirus [25]. In BmCPV the sequence KDLS is partially conserved as KILE and recent cryo-electron microscopic studies of transcribing BmCPV has shown that Lys^234^ is the only lysine found near the GMP moiety to form phosphoamide bond [9]. In AmCPV mutation of the Lys^21^ residue to Ala^21^(K21A) of KLRS motif by site directed mutagenesis showed complete loss of the autoguanylylation activity (Figure 3B, lane 1). These results suggest that KDLS sequence is conserved in AmCPV as KLRS and its lysine^21^ may contributes to the formation of phosphoamide bond. It also indicates that location of lysine residue in RNA GTase participating in the formation of phosphoamide bond with GMP not only differs among the members of the *Reovirodae* family but also among the Cypoviruses.

To understand the role of two histidine residues located at 93 and 105 positions in Hx_n_H motif of AmCPV S5, these were mutated to alanine residues. Mutation of His^93^ to Ala^93^ (H93A) and His^105^ to Ala^105^ (H105A) also completely abolished autoguanylylation activity of p65 (Figure 3B, lanes 3 and 5). These results suggest that histidine at position 93 and 105 are also critical residues for autoguanylylation activity. It has been reported earlier that protonation of two histidine residues (223 and 232) in the GTase domain of orthoreovirus lambda 2 and aquoreovirus is necessary for the GTase activity [23]. Cryo-electron microscopic study have shown that in BmCPV VP3 the corresponding two histidines at position 208 and 217 in HxnH motif are located adjacent to the GMP moiety [9]. It has been suggested that protonation of these two histidine residues makes them positively charged and increases the affinity of GTP for GTase by neutralizing the negative charge of the GTP phosphate group and results in increased GTase activity [9,23]. We hypothesized that in AmCPV p65, in a similar way His^93^ and His^105^ in HxnH motif may enhance the GTase activity.

Effect of pH, temperature and divalent ions in the autoguanylylation showed that pH 5.0 (Figure 3C), temperature 25–30°C (data not shown) and 10 mM MgCl_2_ were optimum for autoguanylylation activity (Figure 3D). Biochemical and kinetic studies on an RNA capping enzyme of *Chlorella* virus PBCV-1, demonstrated that magnesium ion is required for the capping enzyme to efficiently catalyze GMP transfer [32,33]. Recent studies on eukaryotic mRNA guanylyltransferase using empirical and thermodynamic integration p*K*_a_ estimates, along with conventional molecular dynamic simulations also showed that magnesium binding likely activates the lysine nucleophile by increasing its acidity and by biasing the deprotonated nucleophile into conformations conductive to intermediate formation [34]. In a likewise manner the acidic pH and magnesium ions may be helping AmCPV p65 for its maximum GTase activity.

In order to determine the nature of the p65-GMP linkage, the radiolabelled p65-GMP complex was treated with different chemicals and analysed by thin layer chromatography (TLC). The TLC analysis showed that the complex was resistant to alkali treatment and remained at the origin (Figure 3E, lane 2). Treatment with hydroxylamine and hydrochloric acid cleaved the complex, and released the GMP moiety (Figure 3E, lane 3 and 4). These results indicate formation of alkali resistant phosphoamide linkage between p65 and GMP.

### Transfer of guanyl moiety by p65 to 5′ end of viral RNA

**Figure 4:**
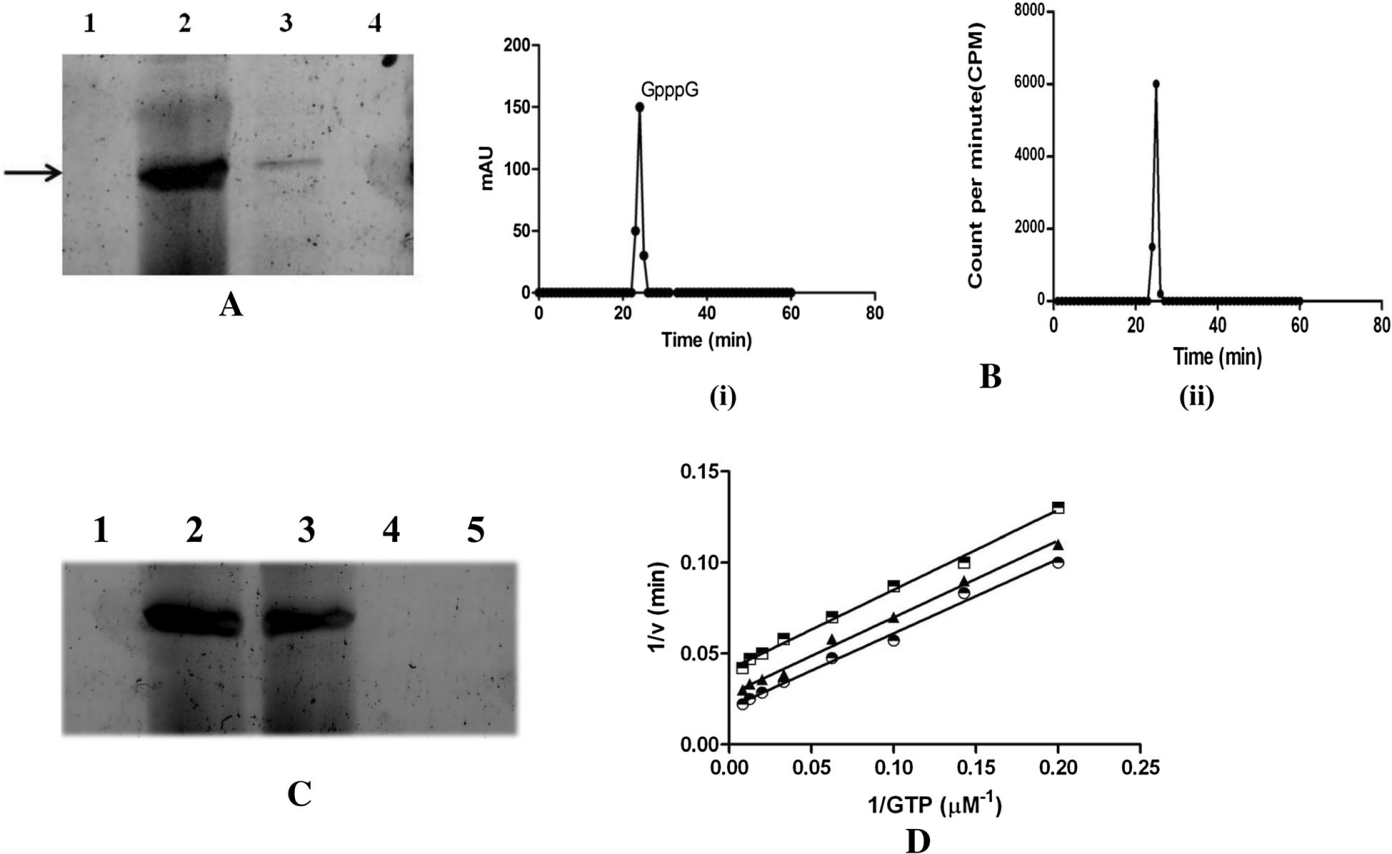
**Biochemical and kinetic analysis of RNA capping. (A)** Analysis of capped RNA by denaturing PAGE followed by autoradiography. Lane 1, no RNA; lane 2, RNA reacted with purified p65; lane 3, RNA reacted with virion and lane 4, no protein. Arrow indicates the position of the radiolabelled RNA band. **(B)** Analysis of only GpppG (i) and nuclease P_1_ resistant labeled capped RNA (ii) by HPLC. The peak indicated the position of GpppG. **(C)** Transfer of labelled GMP moiety by p65 to a di phosphate ended viral RNA. Lane 1, 5' triphosphate ended RNA initiating with ‘G’ base; lane 2, 5' di-phosphate ended RNA initiating with ‘G’ base; lane 3, di-phosphate ended RNA initiating with ‘ A’ base; lane 4, 5' tri phosphate ended RNA initiating with ‘A’ base; lane 5, BSA as negative control. Arrow indicates the position of the respective band. **(D)** Analysis of p65-GTP interaction reaction kinetics. Double reciprocal plot showing the activity of p65 as a function of GTP concentration in the presence of 50 nM (), 100 nM (▲) and 150 nM () RNA.

To gain stronger evidence that p65 possesses GTase activity for the capping of RNA, a transguanylylation activity assay (i.e. transfer of GMP to mRNA) was performed using di phosphate ended AmCPV RNA [35]. Initially the substrate RNA (5′UTR of AmCPV S2) was synthesized by *in vitro* transcription [15] and treated with recombinant 5′ RNA triphosphatase enzyme [obtained by expressing S4 of AmCPV in *E. coli* (unpublished)] to remove the terminal γ phosphate from the 5′ end. Incubation of these transcripts with purified p65 or virion in the presence of [α-^32^P]GTP and analysis of product by denaturing 10% PAGE followed by autoradiography showed generation of ^32^P labelled RNA in samples containing p65 (Figure 4A, lane 2) or virions (Figure 4A, lane 3) while samples lacking p65 (Figure 4A, lane 4), or RNA (Figure 4A, lane 1) did not produce any labelled RNA band. These results indicate that both autoguanylylation and transguanylylation activities are present in purified p65 without any triphosphatase activity. To determine the exact cap structure, the ^32^P labelled capped RNA was digested with nuclease P_1_ and analysed through HPLC along with GMP, GDP, GTP, GpppG marker followed by measurement of radioactivity in a scintillation counter. The nuclease P_1_ resistant products were eluted at the same position as the GpppG marker [Figure 4B (i) and (ii)] indicating the modification (addition of GpppG) of the 5′ end of the *de novo* RNA transcripts by p65.

### Specific trans guanylylation of the A/G ended viral RNA by p65

In order to investigate the preference of p65 for the transfer GMP to in vitro transcribed and triphosphatase treated UTR having ‘A’ or ‘G’ residue at their 5′ end, ppA-RNA and ppG-RNA were incubated with [α-^32^P] GTP in presence of p65. Analysis of reaction products in 10% denaturing polyacrylamide gel followed by autoradiography showed the transfer of label to the both RNA ends in almost equal efficiency (Figure 4C, lane 2 and 3) indicating an equal preference of enzyme (p65) to transfer GMP to ppA-RNA or ppG-RNA. No signal was found in case of triphosphate ended RNA initiating with A/G residue (Figure 4C, lane 1 and 4) indicating that the GMP moiety was transferred to the di phosphate ended A/G RNA but not to triphosphate ended RNA. Therefore, it seems autoguanylylation and transguanylylation by p65 occurs through bi substrate mechanism as observed in case of flavivirus by its NS5 protein [35].

In case of orthoreovirus, it has been reported that all the ten segments can be transcribed simultaneously and the transcripts are released through turret like projection at icosahedral vertices after being capped at the 5′ end [36,37]. In case of BmCPV simultaneous transcription, capping and release of mRNA from the capsid occurs and turret protein has been reported to be involved in this process having both triphosphatase and GTase activity [7]. Further experiments are required to understand involvement of other proteins along with p65 to complete the capping process of AmCPV mRNA.

### Determination of Kinetics parameter for p65

Since p65 catalyzes both autoguanylylation (p65–GMP) and transguanylylation (i.e. transfer of GMP moiety to RNA) reactions during capping of 5′ end of RNA, the bi-substrate reaction kinetics was assayed by varying the concentration of GTP and RNA [38]. The progress of the reaction was monitored with the increase in absorbance at 630 nm as a consequence of the release of inorganic phosphate (Pi) from the bi-product pyrophosphate. A Lineweaver-Burk plot was obtained by plotting these data and showed clearly parallel lines (Figure 4D) providing strong evidence for ping-pong mechanism [33] and not for the quaternary complex formation. Re-plotting these data, the K_m_ for GTP and RNA were obtained as of 34.24 μM and 98.35nM respectively, and K_cat_ was calculated as 540 S^-1^. These results indicate a simple 1:1 interaction between GTP and p65 or RNA with high affinity. Similar type of experiments performed in *Paramecium bursaria Chlorella* virus have determined the K_m_ of this viral GTase as 10.5 and 3.6 um, for GTP and RNA, respectively and these values are very close to that of AmCPV GTase.

### Determination of thermodynamic parameter

Thermodynamic parameter of ligand binding (guanosine 5′-[γ-thio] triphosphate, a GTP analogue or RNA) to p65 was studied using fluorescence spectroscopy. Binding of GTP to p65 showed a significant modification in intensity of intrinsic fluorescence of the protein. In order to better characterize the nucleotide binding to the protein, the K_d_ value for the p65-GTP or p65-RNA complex was determined by measuring tryptophan fluorescence of the protein after exciting at 295 nm where the emission spectra would arise almost exclusively from tryptophan. Therefore, despite the fact that p65 has 32 tyrosine residues and 8 tryptophan residues (single tryptophan near the active site), the emission spectra is dominated by indole fluorophores. This dominance is due, in part, to the higher molar absorption coefficient of tryptophan and resonance energy transfer from tyrosine to tryptophan.

**Figure 5:**
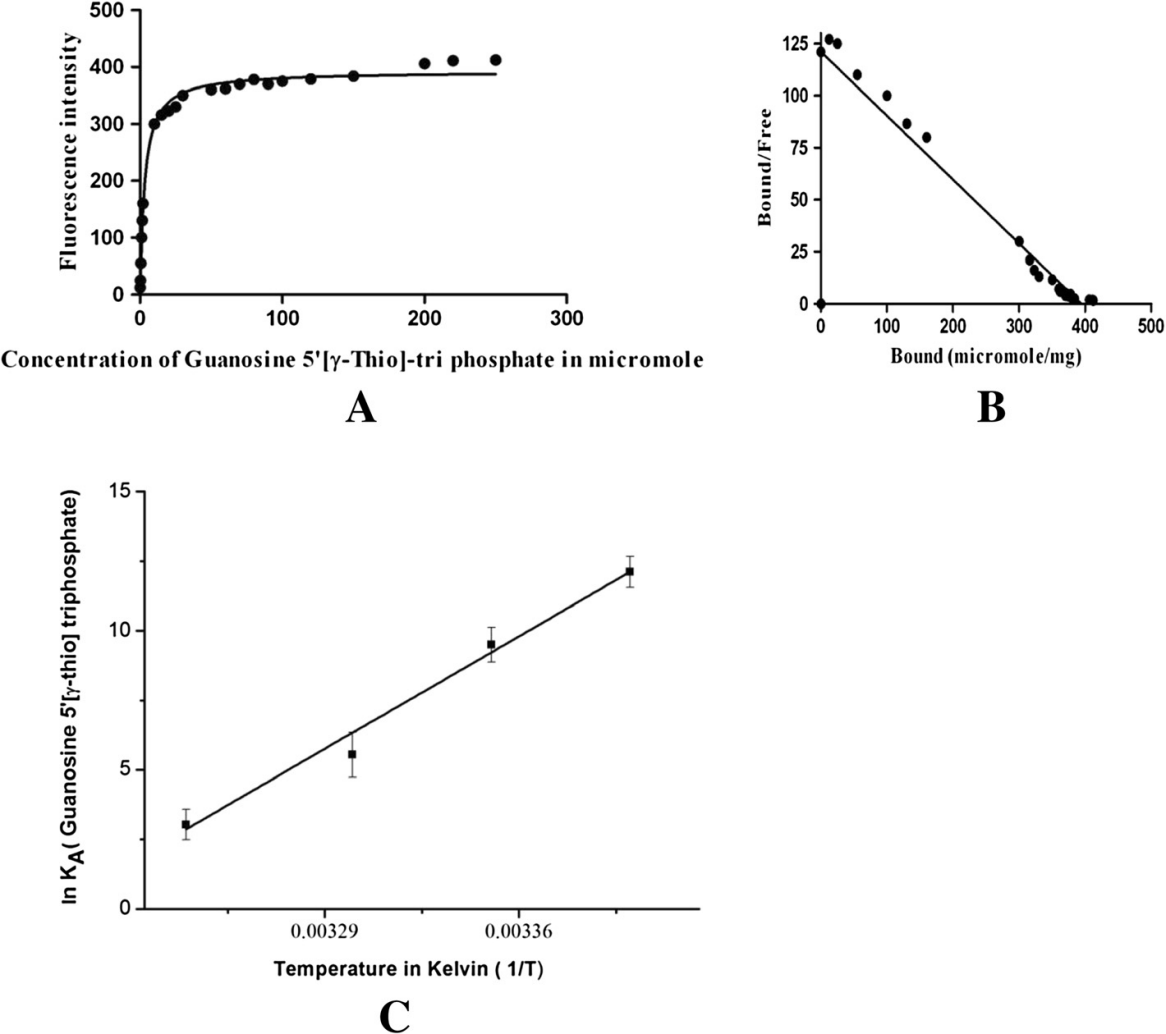
**Analysis of p65 and GTP binding. (A)** Saturation binding curve of guanosine 5' [γ-thio] triphosphate with p65. **(B)** Scatchard plot of binding data of guanosine 5' [γ-thio] triphosphate to p65. **(C)** Van’t Hoff plot for interaction between guanosine 5' [γ-thio] triphosphate analogue and p65 under different temperature at pH 7.0. Values are mean +/- standard error.

**Figure 6:**
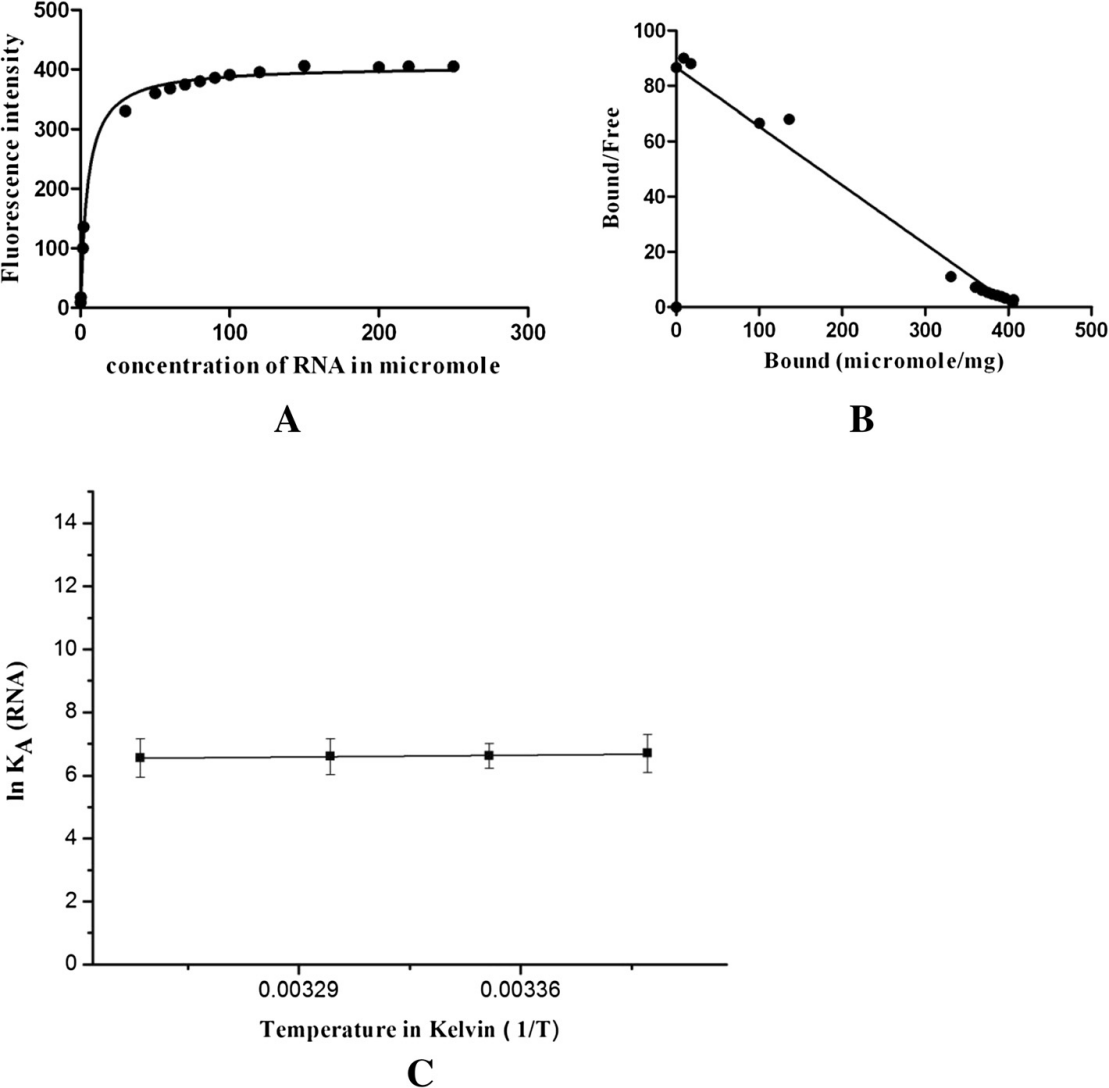
**Analysis of p65 and RNA binding. (A)** Saturation binding curve of p65 with RNA. **(B)** Scatchard plot binding data of RNA to p65. **(C)** Van’t Hoff Plot for the interaction between RNA and p65 protein under different temperature. Values are men +/- standard error.

Fixed concentration of p65 protein was titrated with increasing concentration of guanosine 5′-[γ-thio] triphosphate (GTP analogue) or RNA and the change of fluorescence was recorded. The fluorescence intensity was plotted against the GTP analogue or RNA concentrations to get the saturation binding data (Figure 5A and Figure 6A). As seen in these figures, the binding of GTP analogue or RNA to p65 saturated over micro molar range. Scatchard plots were drawn from these saturation binding data (Figures 5B, 6B) and the K_d_ value for GTP analogue and RNA was calculated as 3.224 × 10^-6^ and 4.6 × 10^-6^, respectively, indicating a strong affinity for ligand.

The affinity of p65 to both GTP analogue and RNA was tested under varying temperature conditions. The Van’t Hoff analysis of these data revealed a liner relationship between natural logarithm of the equilibrium association constant (lnK_A_ = -lnK_d_) and 1/T over the temperature range tested (20°C to 35°C). Though the free energy of binding gives the overall description of a reaction, yet the enthalpy and entropic contribution provides the complete understanding of the driving forces that helps in enzyme-GTP or enzyme-RNA association. Enthalpic and entropic contribution to p65 were calculated from the observed changes in equilibrium constant (K_A_) with temperature. Specifically, estimation of enthalpy (∆H) or entropy (ΔS) associated with p65 were made using the slope and y-intercept, respectively, as described in the Van’t Hoff equation, ln(K_A_) = -(∆H/R) (1/T) + (ΔS/R), where K_A_ is the inverse of K_d_. Changes in the Gibb’s free energy was calculated for each ligand using the equation ∆G = -RT ln (K_A_) at 25°C. The Gibbs energy (∆G) for GTP analogue and RNA were calculated as -3.49 kJ/mol and -6.7 kJ/mol, respectively. The GTP binding step was exothermic at 25°C with a high enthalpy association, (∆H) = -399.1 ± 4.1 kJ/mol with a unfavorable entropic contribution (ΔS = -1.2 ± 0.137) as obtained from Van’t Hoff plot analysis (Figure 5C). These analyses clearly indicate that the initial GTP binding step is primarily driven by enthalpy, with an unfavorable entropic change.

Similarly, the measurement of RNA binding activity of p65 as a function of temperature showed that **A** p65 binding to RNA was clearly dominated by favorable entropic effect (ΔS = 1.7 ± 0.15 kJ/mol), with a minor contribution from a favorable enthalpy change (ΔH = 0.043 ± .0049 kJ/mol) (Figure 6C). Favorable negative enthalpy changes generally associated with contributions of hydrogen bond, Van der Waal’s interaction [39], whereas the unfavorable entropy change is associated with the exposure of hydrophobic surfaces to the surface of the protein or to decrease the conformational flexibility [40].

## Conclusion

AmCPV S5 has been cloned and expressed in bacterial system. Analysis of expressed recombinant protein showed that AmCPV S5 encoded p65 is a bona fide guanylyltrasnferase enzyme that helps to cap the 5′ end of the nascent viral RNA. Kinetic and thermodynamic analysis explains the biochemical and biophysical mechanism of action of this enzyme. These studies will help to understand capping process of cypoviral RNA during viral replication within viral capsid and to find a target for therapeutic application.

## Methods

### cDNA synthesis, molecular cloning and sequencing of AmCPV S5

Purification of AmCPV polyhedra from virus infected larvae was done by following the method of Hayashi and Bird [41] with some modifications [13]. The total dsRNA genome was isolated from purified polyhedra by modified guanidinium isothiocyanate method [42], separated by gel electrophoresis and segment 5 (S5) was eluted by gel extraction kit (Qiagen). Conversion of the S5 ds RNA to its corresponding cDNA was done following the sequence independent RT method [43]. The cDNA was cloned into pCR-XL-Topo vector to create pCR-XL-Topo/AmCPV S5 and after transforming into *E. coli* TOP 10 cells, plasmids were isolated and characterized by *Eco*R1 restriction digestion. Recombinant plasmids containing proper size insert were then sequenced by using Bigdye terminator in an automated DNA sequencer (ABI, model 3100) with M13 forward and reverse primers as well as internal primers designed from deduced sequences.

The sequence of AmCPV S5 was analyzed by sequencher (Gene Codes Corporation) and homology searches were done using BLAST [44]. The molecular weight, isoelectric point and amino acid composition were determined by Protparam of ExPASy (http://www.expasy.ch/tools/protparam.html). Secondary structure was predicted using GOR4 program [45]. Amino acid sequence alignment was done by ClustalW to find the conserve motif among other related viruses and similarity in amino acid sequences was defined using ESPript 2.2 [46] with similarity score 0.8 and physicochemical properties of side chains as criteria for similarity.

### Expression and purification of p65

The entire ORF of AmCPV S5 from nucleotide 266 to 2086 (606 amino acids) was amplified by PCR from plasmid pCR-XL-TOPO/AmCPV S5 using *Accuzyme* DNA polymerase (Bioline) and two synthetic primers AGCPV 182 F (5′-GTTTACTTCAAAG***GGATCC***ATGGTTGG-3′) and AGCPV 183R (5′-GGACAGTTA***CTGCAG***CATATTCGT-3′) containing *Bam*HI (forward primer) and *Pst*I (reverse primer) restriction site (shown underlined). The PCR product was digested with respective restriction enzymes and ligated to *Bam*H1 and *Pst*1 digested pQE-30 vector to create pQE-30/AmCPV S5. Overlapping extension PCR based site directed mutagenesis [47] was done to replace lysine 21 to alanine residue (K21A) in conserve KLRS motif by using two more primers: AGCPV S5 LAF (5′-GTCAAACAATCGTTTAATGCGCTTAGATCT-3′) and AGCPV S5 LAR (5′-AGATCTAAGCGCATTAAACGATTGTTTGAC-3′). Similarly, mutations were created in HxnH motif by replacing both histidine residues with alanine using following primer pairs [AGCPV5HA93F: 5′-CTCTCGATCTCCGCTCCCATATTA-3′) and AGCPV5HA93R: 5′- TAATATGGGAGCGGAGATCGAGAG-3′ for H93A; AGCPV HA105F: 5′-AGGCCCGGCGCCTTGACTGAA-3′ and AGCPVHA105R: 5′- TTCAGTCAAGGCGCCGGGCCT-3′ for H105A]. The resulting recombinant plasmids were then transformed into *E. coli* M15 cells, colonies were screened by restriction digestion and confirmed by sequencing.

For the analysis of recombinant protein expression*, E.coli* bacteria harboring the pQE-30/AmCPV S5 or pQE-30/AmCPV S5 K21A or pQE-30/AmCPV S5 H93A or pQE-30/AmCPV S5 H105A were grown into fresh 5 ml LB media at 37°C till the OD at 600 nm reached to 0.6. Then the culture was induced with 1 mM IPTG for additional 4 h at the same temperature. Bacterial cells were then harvested and analyzed by SDS-10% PAGE [48].

To purify expressed protein (p65) in the soluble form (wild type or mutant), recombinant *E. coli* harboring pQE-30/AmCPV S5 were grown in 1 L LB media, induced with 1 mM IPTG, lysed by sonication in a buffer A (10 mM Tris, 300 mM NaCl pH:8.0) and recombinant protein was purified through Ni-NTA chromatography (Qiagen). The amount of the purified protein was determined by the Bradford method [49] using BSA as standard and the purity was checked by SDS-10% PAGE [48].

### Production of anti-p65 polyclonal antibody

Polyclonal antibody was raised against *E.coli* expressed and purified p65protein in rabbit by standard method [19,50]. Specific antibody was purified by antigen (p65) affinity chromatography [50,51]. The antibody titer of purified antibody was determined by ELISA using 2.5 μg of recombinant p65 antigen.

### Immunoblot analysis of p65 in infected mid gut cell of *A. mylitta* larvae

To determine whether p65 exist as structural or non structural protein in AmCPV, western blot analysis of virion particle, polyhedra, infected and uninfected mid gut of *A.mylitta* larvae along with bacterially expressed recombinant p65 was done using anti-p65 antibody raised in rabbit. The purified polyhedra (0.5 g) was dissolved in 15 ml 0.2 M carbonate buffer (pH 10.2), incubated at 27°C for 1 hr and then neutralized by 0.2 N HCl slowly to pH 7.0. Virion particles were pelleted by ultracentrifugation at 27,000 r.p.m for 15 min at 4°C and washed twice with PBS (pH 7.3) and finally resuspended in PBS. The mid gut of uninfected and AmCPV-infected fifth instars larvae, purified polyhedra, recombinant p65 and virion particle were separated in SDS-10% PAGE gel and then electrophoretically transferred onto nitrocellulose membranes [52]. The membrane was blocked with 3% casein in 1X PBS (0.14 M NaCl, 2.7 M KCl, 0.0018 M KH_2_PO_4_, 0.008 M Na_2_HPO_4_, pH 7.4) for 1 hr at room temperature. After blocking, the membrane was washed with 1X PBS for three times and then incubated with anti-p65 polyclonal antibody (1:100) for 1 hr at room temperature. After washing with 1X PBS, the membrane was incubated with 200 fold diluted Protein A-HRP for 1 hr at room temperature. Then the membrane was washed thoroughly with 1X PBS for three times and color development was done using the HPO color development kit (Bio-Rad).

### Assay for p65 - GMP complex formation

The assay was performed by incubating 1 μg of purified p65 protein with 30 μM of [α-^32^P] GTP in a buffer containing 50 mM Tris -HCl (pH 7.5), 5 mM DTT and 5 mM MgCl_2_ for 30 min at 30°C [36]. Parallel reactions with isolated AmCPV virions (1 μg) and bovine serum albumin (1 μg) were done as positive and negative controls, respectively. The reaction was stopped by addition of 10 mM EDTA and 1% SDS, analyzed by SDS-10% PAGE and autoradiographed in a phosohorImager (GE healthcare) to visualize radio labelled protein band. Assays were also done similarly using mutant (K21A), (H93A) and (H105A) p65 proteins.

To determine the optimum reaction condition, the autoguanylylation reaction was performed in different temperatures (4 to 40°C) and pH (4 to 8). The effect of different divalent ions (Mg^2+^, Mn^2+^, Ca^2+^, Cu^2+^, Ni^2+^) on autoguanylylation was also determined by performing the reaction in presence of different concentrations of these divalent ions (0 to 60 mM). The Mg^2+^, Mn^2+^ and Ca^2+^ were used as chloride salts whereas Cu^2+^ and Ni^2+^ were used as sulfates. After analyzing reaction products on SDS-10% PAGE followed by autoradiography, protein bands were cut from the gel and the amount of labeled p65-GMP protein complex was measured in a liquid scintillation counter (Perkin Elmer).

To examine the nature of exact p65-GMP linkage type, the assay was performed using the same procedure as described for the GTase assay of vaccinia virus and lambda peptide of reovirus [53,54]. After incubation of p65 with [α-^32^P] GTP the covalent radiolabelled p65-GMP complex was treated with 0.5 M NaOH or 0.5 M HCl at 70°C for 15 min, or with 3.8 M hydroxylamine (Merck) in 0.2 M sodium acetate (pH 4.8) at 37°C for 20 min. The reaction products were then analyzed by thin layer chromatography (TLC) using PEI-cellulose plate [55,56], in 0.75 M KH_2_P0_4_. The plate was then exposed to x-ray film and developed.

### Preparation of 5′ ‘A’ or ‘G’ containing diphosphate ended RNA substrate

To analyze the transfer of GMP moiety to a pp-ARNA or pp-GRNA by p65, 5′UTR of AmCPV S2 RNA (~270 bp) was generated having ‘A’ residue to its 5′ end by *in vitro* transcription [15]. In a similar way, using TF7 (5′ GCTCTAGA**TAATACGACTCACTATA**GTTACTAGTAATCATCCTTG-3′) and TF2 (5′ AATCGCGTAATCTGCACTCAC) primers AmCPV S2 RNA transcript having G residue at the 5′ end was also generated by *in vitro* transcription. The terminal phosphate from the 5′ end of these in vitro transcribed RNA was removed by incubating with 1.5 μg of RNA transcript with 1.5 unit of AmCPV S4 encoded recombinant RNA triphophatase (unpublished) in a buffer containing (250 mM Tris–HCl, 100 mM NaCl, 1 mM MgCl_2,_ pH 8.0) at 37°C for 1 hr. Finally, RNA was extracted with phenol: chloroform (1:1), precipitated with ethanol, resuspended in nuclease free water and quantified in a nanodrop spectrophotometer.

### Transfer of GMP to RNA

Both 5′ diphosphate ended RNA transcripts i.e. ppA-RNA and ppG-RNA (1 μg) were incubated in a buffer containing 50 mM Tris–HCl at pH 7.5, 5 mM MgCl_2_, 5 mM DTT, 5 μg of p65, 30 μM [α-^32^P] GTP at 37°C for 30 min. Parallel reactions using no RNA template or p65 were performed as controls. Similarly, experiments were also performed with triphosphate ended RNA transcripts (without recombinant RNA triphosphatase treatment). The reaction products were analyzed on 8 M urea containing 10% polyacrylamide gel and autoradiographed with a phosphoImager (GE healthcare).

### Determination of the cap structure

After the transfer of the labeled GMP moiety to the ppRNA (as described before), the formation of exact cap structure was determined through HPLC followed by scintillation counting [56]. In brief, an aliquot of 20 μl of the labelled reaction product was analyzed on a 8 M urea containing 10% polyacrylamide gel and the remaining 30 μl was digested with nuclease P_1_ (US biological) enzyme at 37 °C for 1 hr. Nuclease P_1_ was removed by phenol-chloroform (1:1) extraction and nuclease P_1_-resistant molecules were precipitated with ethanol and analyzed by HPLC together with of unlabelled GMP, GDP, GTP, GpppG marker.

For HPLC, the reaction mixture was applied to a HPLC anion exchange column (ZORBAX SAX 5 μ from Agilent) and products were eluted with a salt gradient of 0.04 M to 0.5 M KH_2_PO4 (pH 5.5) at a flow rate of 1 ml/min. Fractions were collected every minute and the amount of radioactivity was measured in a liquid scintillation counter (Perkin –Elmer).

### Determination of ligand binding parameter of p65

Initially, the background-corrected maximum emission wavelength of purified p65 [in 50 mM Tris–HCl (pH 7.5) and 50 mM potassium acetate] was determined by exciting the p65 at 295 nm followed by scanning from 310 to 440 nm [11]. After obtaining the maximum emission wavelength of p65, i.e. at 335 nm, all the thermodynamical parameters due to ligand binding (GTP and RNA) were determined at 335 nm. Briefly, increasing amounts of GTP analogues (0.1 μM to 250 μM) and RNA (0.1 μM to 250 μM) were added to a 100 nM solution of the p65 (enzyme) in a binding buffer (50 mM Tris–HCl, (pH 7.5), 50 mM KOAc) at 25°C and the emission spectrum was determined at 335 nm in a fluorescence spectrophotometer. The K_d_ values were determined through scatchard plot analysis from saturation binding isotherm. Additional analysis of the equilibrium binding constant in the various temperature (20 to 30°C) were conducted to determine the thermodynamic parameters for Gibb’s free energy, enthalpy and entropy using the Van’t Hoff plot. This thermodynamic relationship is described by the equation below

(1) $$‒\mathrm{RTln}k_{A}=\mathrm{\Delta G}=\mathrm{\Delta H}‒\mathrm{T\Delta S}$$

(2) $$\ln\left(K_{A}\right)=‒\left(\mathrm{\Delta H}/R\right)\left(1/T\right)+\left(\mathrm{\Delta S}/R\right)$$

R is the gas constant (8.314 J.K^-1^. M^-1^), G is Gibbs free energy, **∆**H and **∆** S are the changes in enthalpy and entropy, respectively and T is the absolute temperature, K_A_ is the association binding constant of the ligand with the enzyme (i.e. 1/K_d_).

### Kinetics analysis

All the kinetic studies of capping reaction were performed at 25°C by monitoring the release of inorganic phosphate (Pi) from pyrophosphate (PPi), a bi-product of the capping reaction, using pyrophosphatase enzyme (Fermentas). Then the released Pi was measured in spectrophotometer at λ _630_ nm on the basis of the principle that initially the Pi reacts with ammonium molybdate in acidic medium and forms phosphomolybdate complex. Then the malachite green reacts with phosphomolybdate complex in presence of Tween 20 at lower pH to produces a green color complex. The concentration of the Pi is directly proportional to absorption maxima at 630 nm [57].

In brief, varying concentration of GT (0 to 120 μM) and diphosphate ended RNA (50 nM, 100 nM, 150 nM) were incubated with constant amount of p65 (0.5 μg ) in a buffer containing 50 mM Tris–HCl (pH 7.5), 5 mM DTT and 5 mM MgCl_2._ The PPi, bi –product of the reaction was cleaved into Pi by one unit of inorganic pyrophophatase (NEB) at 37°C for 1 hr and the amount of Pi released was calculated from prepared standard curve of Pi generated from di sodium hydrogen phosphate by the same procedure. Kinetic parameters like apparent K_m_, apparent V_max_ were determined by direct fit of rate versus substrate concentration data to Michaelis-Menten equation (using Graph pad Prism 4.0 software) followed by the Lineweaver burk plot for each of RNA concentration. The exact K_m_ and V_max_ were determined by replotting the apparent K_m_ and apparent V_max_ values.

### Nucleotide sequence accession number

The nucleotide sequence of AmCPV S5 has been deposited in the Genbank database under Accession no: JX853836.

## Competing interests

The author declares that they have no competing interests.

## Authors’ contribution

PB designed the research study, performed the experiments, analysed the data and contributed to the writing of the manuscript. AK helped in bioinformatics analysis of sequence data. AKG supervised the whole work and contributed to the writing of the manuscript. All authors read and approved the final version of the manuscript.
